# Understanding and manipulating plant lipid composition: Metabolic engineering leads the way

**DOI:** 10.1016/j.pbi.2014.04.001

**Published:** 2014-06

**Authors:** Johnathan A Napier, Richard P Haslam, Frederic Beaudoin, Edgar B Cahoon

**Affiliations:** 1 Department of Biological Chemistry, Rothamsted Research, Harpenden, Herts, AL5 2JQ, UK; 2 Center for Plant Science Innovation and Department of Biochemistry, University of Nebraska-Lincoln, Beadle Center, 1901 Vine St, Lincoln, NE 68588, USA

## Abstract

The manipulation of plant seed oil composition so as to deliver enhanced fatty acid compositions suitable for feed or fuel has long been a goal of metabolic engineers. Recent advances in our understanding of the flux of acyl-changes through different key metabolic pools such as phosphatidylcholine and diacylglycerol have allowed for more targeted interventions. When combined in iterative fashion with further lipidomic analyses, significant breakthroughs in our capacity to generate plants with novel oils have been achieved. Collectively these studies, working at the interface between metabolic engineering and synthetic biology, demonstrate the positive fundamental and applied outcomes derived from such research.

## Highlights

• Description of recent advances in plant lipid metabolism.
• Description of break-through achievements in plant metabolic engineering.
• Insights into the practical applications of plant synthetic biology.

**Current Opinion in Plant Biology** 2014, **19**:68–75

This review comes from a themed issue on **Physiology and metabolism**

Edited by **Sarah E O’Connor** and **Thomas P Brutnell**

For a complete overview see the Issue and the Editorial

Available online 15th April 2014

**http://dx.doi.org/10.1016/j.pbi.2014.04.001**

1369-5266/© 2014 The Authors. Published by Elsevier Ltd. This is an open access article under the CC BY license (http://creativecommons.org/licenses/by/3.0/).

## Introduction

Plant seeds play a vital role in human life, providing multiple sources of food and fuel. This is predominantly derived from the storage compounds (oil, protein and carbohydrate) that the developing seed accumulates as energy reserves for catabolism during germination. The ability to harness and use these storage compounds has historically underpinned the transition from hunter-gather to agricultural-based society, and now continues to feed the ever-increasing global population. The predominant storage oil in seeds are neutral lipids such as triacylglycerol and given their significance for nutrition and industry, considerable effort has focussed on the desire to improve both the composition and yield of vegetable oils. However, the apparent simplicity of a seed (as the inert container of useful storage reserves) is misleading, since there is still much to learn about how these compounds are co-ordinately synthesized and compartmentalised. On the other hand, our ability to manipulate and tailor the composition of these reserves is steadily increasing, driven forward by advanced plant metabolic engineering/synthetic biology and informed by detailed metabolite analyses. The adoption of such multidisciplinary approaches has extended our understanding of seed lipid metabolism and, as will be discussed below, generated new specialized platforms for oil production. Indeed, progress in vegetable oil production is now extending beyond the seed and exciting insights are emerging about the possibility of oil production in vegetative tissue. Given the pressures on the carbon economy, exemplified by an increased demand and a declining supply of conventional fossil oil, progress in meeting the requirements for vegetable oil is timely. In this article, we will focus on the synthesis and manipulation of one specific type of storage reserve — triacylglycerol – since this represents one of the best examples of complex metabolic engineering in transgenic plants.

## Development of crop metabolic engineering platforms for translation of specialty oil traits

Over the last two decades, the challenge for researchers has been the accumulation of novel fatty acids, which have beneficial functional groups or properties, into oilseeds with good agronomical traits. Although, metabolic engineering of oil-related traits has largely relied principally upon Arabidopsis as a host to test individual genes and gene combinations for modifying seed oils and more recently for engineering of oil production in vegetative tissues as described below. As proof of principal has been established in Arabidopsis, interest has grown in translation of these oil traits in established oilseed crops. For specialty oils, including those enriched in fish oil-type long chain polyunsaturated fatty acids (LC-PUFAs) and industrially valuable unusual fatty acid structures, attention has centred on non-food oilseed crops to mitigate the unintended mixing of food and specialized oil traits. Camelina (*Camelina sativa*) has emerged as a particularly attractive metabolic engineering host because it can be readily transformed using an *Agrobacterium*-based floral infiltration method [1]. With a relatively short-life cycle, complex metabolic engineering involving the stacking of numerous pathway genes is therefore feasible in Camelina [2^•^], as described below for LC-PUFA engineering. To facilitate genetic improvement of Camelina, a developing seed transcriptome was recently generated, and its utility was demonstrated by its use for engineering a high oleic acid oil trait for improved oil oxidative stability [2^•^]. This was achieved by the seed-specific RNAi suppression of *FAD2* (Fatty Acid Desaturase 2) that controls Δ12 desaturation of oleic acid and *FAE1* (fatty acid elongase 1) that mediates oleic acid elongation to C20 and C22 chain lengths [2^•^]. In addition to the use of Camelina, interest has arisen in Crambe (*Crambe abyssinica*) as a dedicated industrial oil crop, and the recent development of an *Agrobacterium*-mediated transformation system has enabled its use for metabolic engineering of seed oil traits [3]. As proof-of-principal efforts progress to engineer oil production in Arabidopsis leaves, the need for transformable biomass crops, such as sweet sorghum, will be required for translation of this trait.

## Nutritional enhancement of seed lipids — omega-3 polyunsaturated fatty acids

Virtually all plant seeds contain storage lipids in the form of triacylglycerol (TAG). As the terminal point in seed oil biosynthesis, TAG is comprised of a glycerol backbone onto which three fatty acids are sequentially esterified. Plant oils are rich in C18 fatty acids, including the essential fatty acids linoleic acid (18:2Δ9,12,n−6; LA) and α-linolenic acid (18:3Δ9,12,15,n−3; ALA), but are devoid of LC-PUFAs, such as arachidonic acid (20:4Δ5,8,11,14, n−6; ARA), eicosapentaenoic acid (20:5Δ5,8,11,14,17,n−3; EPA) and docosahexaenoic acid (22:6Δ4,7,10,13,16,19,n−3; DHA), which typically only enter the human diet as oily fish. The health benefits of the omega-3 LC-PUFAs EPA and DHA are now well-established [4], and the omega-6 ARA is important for infant nutrition [5]. Given the desire for a sustainable supply of LC-PUFA, efforts have focussed on enhancing the composition of vegetable oils to include the essential LC-PUFAs. The omega-3 forms, specifically EPA and DHA, have been targeted with the ultimate goal of producing a terrestrial plant-based source of these so-called fish oils. Although historically considerable effort has been expended towards this goal (e.g. [6–10], reviewed in [11,12^•^]), efficient modification of seed oil profiles to include these non-native fatty acids has until recently met with limited success. This is despite the early functional characterisation of all the genes required for the primary biosynthesis of EPA and DHA from a range of lower eukaryotes such as algae, diatoms and oomycetes [12^•^,13]. Latterly, two different approaches have shown important advances, both focussed on overcoming the inherent metabolic bottlenecks previously identified as rate-limiting in the heterologous reconstitution of this pathway in transgenic plants [12^•^]. Petrie *et al.* [14] first developed a leaf-based transient expression system to identify a set of omega-3 LC-PUFA biosynthetic genes with high enzyme activities and desired substrate (acyl-CoA) preference. They also co-expressed the master seed regulator WRI1, resulting in the ectopic expression of seed-specific metabolic pathways and the synthesis of seed storage reserves such as TAG, but also facilitating the expression of these omega-3 LC-PUFA transgenes under the control of seed-specific promoters. The utility of this approach allowed for the rapid validation of seed-specific constructs, which would otherwise be dependent on stable transformation [14]. With this knowledge the authors were then able to assemble a large T-DNA construct for stable seed-specific transformation of Arabidopsis, and reported a high level of DHA (but not EPA) in seed oil [15^•^]; a similar approach yielded lines accumulating significant ARA [16]. In an alternative approach to the identification of optimal enzyme activities, Sayanova *et al.* [17] used heterologous yeast expression combined with acyl-CoA profiling to select efficient activities, which were then validated by stable expression in Arabidopsis and camelina. A systematic study was then carried out to identify preferential combinations of biosynthetic enzymes (desaturases and elongases), resulting in the evaluation of 12 different constructs (of 3–7 transgenes) in Arabidopsis [18^•^]. The efficacy of each enzyme combination was validated using lipidomic analysis to inform each subsequent iteration. Using this approach, the authors were able to show a 10-fold increase in the accumulation of EPA [15^•^]. Collectively, these recent studies demonstrate that in the case of the model Arabidopsis, accumulation of significant (meaning similar to that found in fish oils) levels of EPA or DHA is now achievable. Recently, Camelina seed oil was engineered to accumulate EPA and DHA [19^••^] — in this study, the authors report the highest levels of C20+ omega-3 LC-PUFAs in a *recognised* oilseed crop — 31% EPA or 25% EPA plus DHA. This represents not only a new source of fish oils, but a significant demonstration of the power of plant metabolic engineering to overwrite endogenous lipid metabolism.

## Making industrial oils in seeds

**Figure 1:**
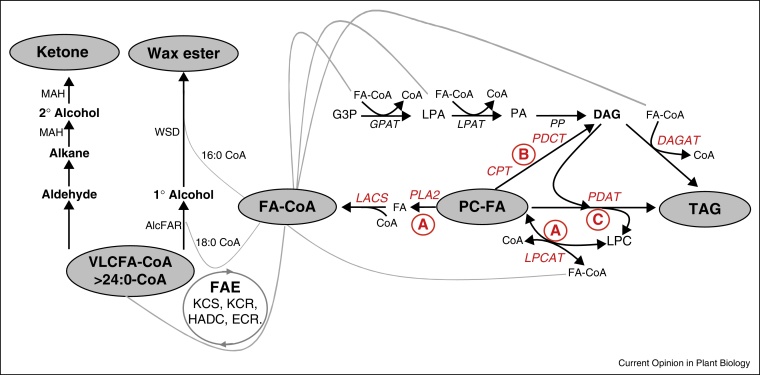
Schematic diagram of the main lipid classes and biochemical pathways involved in the production of TAG and other lipids in developing seeds. There are three mechanisms for the removal of LC-PUFA from PC to make it then available for incorporation into TAG (mechanisms A, B, and C) — please see [23^•^] for full description. For mechanism A, FAs esterified to phosphatidylcholine (PC) (such as FAD2-like products) are under a constant dynamic exchange with the acyl-CoA pool in a process described as acyl editing. Removal of FAs from PC can proceed by the reverse action of acyl-CoA:lysophosphatidylcholine acyltransferase (LPCAT) or the combined action of phospholipase A2 PLA_2_ and long-chain acyl-CoA synthetase, LACS. Once in the acyl-CoA pool, acyl-CoAs and glycerol-3-phosphate (G3P) can be converted into TAG by the consecutive action of acyl-CoA:glycerol 3-phosphate acyltransferase (GPAT), acyl-CoA:lysophosphatidic acid acyltransferase (LPAT), phosphatidic acid phosphatase (PAP), and acyl-CoA:diacylglycerol acyltransferase (DGAT). For mechanism B, the PC head group can be removed, producing a DAG molecule containing the same FAs. This reaction can proceed by four enzymatic mechanisms: phospholipase C, phospholipase D along with PAP, the reverse action of CDP-choline:diacylglycerol cholinephosphotransferase (CPT), or the recently identified phosphatidylcholine:diacyglycerol cholinephosphotransferase, (PDCT). The DAG produced by these mechanisms can then be utilized to produce TAG. For mechanism C, direct transfer of the *sn*-2 FA of PC to the *sn*-3 of DAG produces TAG via a phospholipid:diacylglycerol acyltransferase (PDAT). In addition, the acyl-CoA pool generated by mechanism A or resulting from direct export from the plastid can be accessed by additional enzymes such as the endogenous FAE elongase system to generate very long chain fatty acids (VLCFAs) or heterologous activities such as those involved in wax ester or ketone biosynthesis.

A long-term goal of oilseed metabolic engineering has been the generation of fatty acid traits targeted for industrial applications. A particular focus has been the transfer of biosynthetic and metabolic pathways for unusual fatty acids, such as hydroxy and epoxy fatty acids (used for lubricants, nylon precursors, and plasticizers) from non-agronomic plant species to existing oilseed crops. After more than a decade of gene discovery efforts and numerous basic and translational breakthroughs [20], many challenges remain for achieving levels of unusual fatty acid accumulation in engineered oilseeds that approach the high levels typically found in seeds of non-agronomic gene source species. This is particularly true for metabolic pathways involving the production of unusual fatty acids from functionally divergent Δ12 desaturases (or, FAD2). The most studied of these is the pathway for production of ricinoleic acid (12 OH-18:1Δ9) and related C18-C22 omega-6 hydroxylated fatty acids. These fatty acids are generated by variant FAD2 hydroxylases that principally use oleic acid (18:1Δ9) bound to phosphatidylcholine as a substrate. Castor bean (*Ricinus communis*), which has limited commercial cultivation because of the high content of ricin toxins in it seeds, accumulates ricinoleic acid to 90% of the fatty acids of its seed oil through this pathway. To date, the transfer of the castor FAD2-related hydroxylase together with specialized castor acyltransferases, including the castor diacylglycerol acyltransferase 2 (DGAT2) and phospholipid-diacylglycerol acyltransferase 1 (PDAT1), have yielded only 20–30% ricinoleic acid and other derivative hydroxy fatty acids in transgenic Arabidopsis seeds [21,22]. Results from recent labeling studies of Arabidopsis seeds engineered to express the castor bean hydroxylase indicated an inefficiency in diacylglycerol (DAG) flux through phosphatidylcholine (PC) following oleate hydroxylation for the formation triacylglycerol containing hydroxylated fatty acids (summarised in Figure 1) [23^•^]. Consistent with this, the Arabidopsis *rod1* mutant defective in phosphatidylcholine:diacylglycerol cholinephosphotransferase (PDCT)-mediated flux of DAG through PC, displayed reduced hydroxy fatty acid synthesis in seeds engineered for castor bean hydroxylase expression [24]. The substitution of Arabidopsis PDCT with the castor bean PDCT in this background yielded increased hydroxy fatty acid accumulation, demonstrating that a specialized castor bean PDCT activity is necessary for high level hydroxy fatty acid accumulation [24].

Defective flux of acyl chains from PC to TAG in engineered seeds appears to be a common bottleneck for the accumulation of unusual fatty acids. This was previously noted for conjugated fatty acid accumulation in Arabidopsis and soybean seeds engineered to express FAD2-related fatty acid conjugases that convert either the Δ9 or Δ12 double bond of linoleic acid linked to PC into two conjugated double bonds, which enhances the drying properties of vegetable oils [25]. More recently, studies with the *Escherichia coli* cyclopropane synthase illustrated how the transgenic expression of this enzyme in Arabidopsis seeds effectively converted the Δ9 double bond of oleic acid into a cyclopropane ring, a result which confers vegetable oils with a wide range of industrial functionalities [26]. This reaction uses oleic acid bound primarily to the *sn*-1 position of PC as a substrate [26]. The seed-specific co-expression of the *E. coli* cyclopropane synthase with a lysophosphatidic acid acyltransferase (LPAT) from *Sterculia foetida* seeds, which naturally accumulate high levels of cyclopropane fatty acids, resulted in small increases in cyclopropane fatty acid accumulation. LPAT catalyzes the acylation of the *sn*-2 position of the glycerol backbone in the TAG biosynthetic pathway. Consistent with this activity, increased amounts of cyclopropane fatty acids in TAG in the engineered Arabidopsis seeds was due primarily to enhanced accumulation at the TAG *sn*-2 position [26]. Despite this, cyclopropane fatty acid levels were disproportionately high in PC, accounting for >40% of the PC fatty acids compared to ∼9% of the TAG fatty acids [26]. These results underscore the significance of the metabolic bottleneck for flux of unusual fatty acids into TAG following their synthesis on PC as a major limitation for producing industrial fatty acids in engineered oilseeds.

Recent efforts to produce oils with industrial functionality have also targeted pathways that use only acyl-CoA substrates to produce novel oils, bypassing the intricacies of unusual fatty acid biosynthetic pathways involving PC-linked substrates. One such pathway is that for wax ester biosynthesis. Wax esters lack diacylglycerol backbones and consist of a fatty acid linked through an ester bond to a fatty alcohol. These molecules are desirable for use as high temperature lubricants and are synthesized in a two-step biosynthetic pathway involving conversion of a fatty acyl-CoA to a fatty alcohol via a fatty alcohol reductase (FAR) and condensation of the fatty alcohol with an acyl-CoA via a wax synthase (WS). Recently, Heilmann *et al.* demonstrated the feasibility of co-expressing an endoplasmic reticulum (ER)-localized mouse WS with a mouse peroxisomal FAR retargeted for ER localization to generate wax esters in Arabidopsis seeds with principally C18 and C20 fatty acid and fatty alcohol components [27]. By linking theses enzymes at their amino-termini to oleosin, an oil body structural protein, and fluorescent protein tags, wax ester contents as high as 45 μg/mg seed weight or ∼15% of the total seed oil were achieved [27]. In addition, wax esters highly enriched in oleoyl alcohol and oleic acid moieties were obtained by expression of the mouse enzymes in an Arabidopsis *fad2/fae1* mutant that has high levels of oleic acid in its seeds [27].

Metabolic engineering of very long-chain fatty acid production also offers an opportunity for generating industrial oils through acyl-CoA reactions that bypass PC-linked biosynthetic pathways. Crambe seed oil is naturally enriched in erucic acid (22:1; ∼60% of the total oil), a C22 monounsaturated fatty acid [28^•^]. This fatty acid is a precursor of erucamides, which are slip agents in polyethylene film. To address this need for high-erucic acid vegetable oils, a newly developed transformation protocol was used for introduction of three transgenes with seed-specific promoters: FAD2 RNAi transgene to increase oleic acid content; *Brassica napus* FAE1 to enhance elongation of oleic acid to erucic acid; and a specialized *Limnanthes douglasii* LPAT to increase erucic acid incorporation into the *sn*-2 position of TAG [12^•^]. The result of this multi-gene engineering effort was an increase in erucic acid of up to 73% of the oil in the top performing lines [28^•^]. Additional analyses of these seeds using radiolabeling indicated that compared to other oilseeds, including safflower (*Carthamus tictorius*) seeds, Crambe seeds are particularly effective at producing high levels of erucic acid through acyl-CoA reactions, due to a low PDCT activity that effectively precludes exchange of fatty acids between DAG and PC [29^•^]. Labeling studies of the engineered crambe seeds at different developmental stages revealed that the majority of erucic acid is synthesized at later stages of seed development. Based on this finding, enhanced erucic acid production could be achieved by engineering initiation of biosynthetic and metabolic pathways for erucic acid at earlier seed development stages [29^•^].

## Oil production in green biomass: metabolic engineering of high oilseed-like triacylglycerol accumulation in vegetative tissues

The pressing need to produce more energy from plant biomass has encouraged attempts to produce oil in vegetative tissues. Although seeds and some fruit pericarps (e.g. oil palm, olive and avocado) are by far the largest source of plant produced oils, many other tissues are capable of synthesizing triacylglycerols and a number of studies have reported the presence of cytosolic lipid droplets in leaf mesophyll cells [30]. TAGs notably accumulate during senescence in leaves, under stress and in Arabidopsis mutants disrupted in ER to chloroplast lipid trafficking. Nevertheless, the oil content of vegetative tissues is typically very low in the majority of plant species [31^••^,32].

The possibility of producing TAGs for biodiesel in leaves and other vegetative tissues has recently attracted considerable interest [33]. A number of studies have demonstrated that TAG accumulation can be increased by ectopic expression of individual biosynthetic enzymes such as acyl CoA:diacylglycerol acyltransferase (DGAT) or monoacyglycerol acyltransferases MGAT [34,35^•^], transcription factors such as LEAFY COTYLEDON1 (*LEC1)*, *LEC2* or *WRINKLED1* (*WRI1*) [36,37,38] that control seed development and maturation, or by mutating genes involved in TAG and fatty acid turnover such as COMATOSE (*CTS2*), SUGAR DEPENDENT1 (*SPD1*) or COMPARATIVE GENE IDENTIFICATION-58 (*CGI58*) [31^••^,39,40]. However, in most of these studies increases in TAG leaf content was only very modest and/or dependent on the supply of carbohydrates. Since key enzymes for both oil synthesis and breakdown are expressed in vegetative tissue it was suggested that achieving substantial levels of storage lipid in leaf biomass required the re-orientation of carbon flux into TAG, as indicated by the additional effect observed when overexpressing *LEC2* in the *cts2* β-oxidation mutant [39,41^••^]. Recently, several groups have reported improved oil accumulation in leaves by modifying the expression of gene pairs i.e. combinations of either *WRI1* or *LEC2* [32,34] or an engineered oleosin [42] with DGAT1 or PDAT [43]. However, dramatically increased TAG levels (exceeding 15% of dry weight in vegetative tissue) have only been achieved via integrated metabolic approaches (so-called ‘Push, Pull and Protect’) enhancing fatty acid and TAG synthesis while preventing lipolysis [31^••^,41^••^]. Latterly, the identification of non-seed proteins involved in the binding and stabilization of lipid-rich particles in the cytosol of plant cells [44] has identified a new aspect of the cellular machinery regulating the packaging of triacylglycerol's in plant vegetative tissue.

It will be interesting to investigate whether oil accumulation in green biomass can be further improved without severely impacting photosynthesis and plant development. One possibility for achieving this could be the use of senescence induced promoters to engineer plants in which TAG accumulation is initiated only after leaves have reached their maximum size [33]. Another might directly connect carbon fixation to fatty acid biosynthesis; introducing a functional glycolytic pathway converting 3-phosphoglycerate to phosphenolpyruvate. Whichever possibility is adopted, the goal of using photosynthetic cells to accumulate very high levels of oil is attractive. However, matching the accumulations seen in seeds able to accumulate more than 35% TAG (% of dry weight) remains a formidable metabolic engineering challenge.

## Conclusions

**Figure 2:**
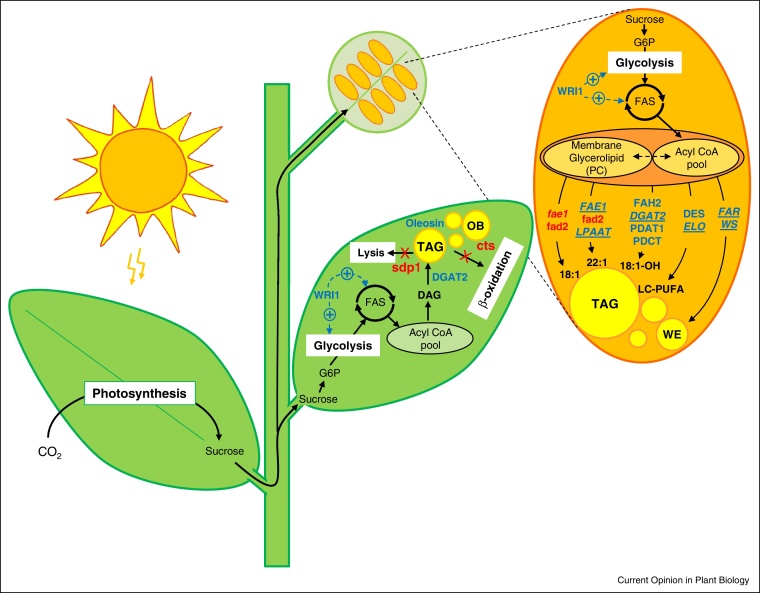
Schematic representation of metabolic engineering strategies for manipulation of oil content and composition in vegetative and seed tissues. Different approaches described in this article are highlighted. Blue: target genes suitable for overexpression; Red: target genes for inactivation by mutation or RNAi constructs. Genes encoding enzymes using acyl-CoA substrates are underlined. FAS = plastid localised fatty acid synthase

The engineering of economically viable levels of LC-PUFAs in camelina seeds and ‘ultra-high’ levels of erucic acid in crambe seeds represent recent successes in the translation of specialty fatty acid traits to oilseed crops. The ability to achieve high amounts of unusual fatty acid production by transfer of PC-linked biosynthetic and metabolic pathways from seeds of non-agronomic species to seeds of either the Arabidopsis model or existing oilseed crops remains elusive to metabolic engineers. Solving bottlenecks that limit the synthesis and accumulation of these fatty acids will require more in-depth understanding of fatty acid metabolic pathways in seeds that naturally accumulate high levels of unusual fatty acids. It will also be necessary to determine the relative contributions of different enzymes specialized for these pathways in the native species and to possibly down-regulate non-productive, competing pathways in seeds of host oilseeds- this is summarised in Figure 2. The integrated approach of engineering transcription factors that up regulate fatty acid synthesis and overexpression of TAG biosynthetic enzymes to sequester the enhanced fatty acid production coupled with downregulation of TAG catabolic enzymes is proving to be an effective strategy for generating substantial levels of oil in leaves of model plants. Successful translation of these strategies in existing biomass crops such as sweet sorghum will likely also require the selection of promoters for transgenes that allow the persistence of accumulated oil through leaf senescence. Future success of metabolic engineering of specialty oil traits will likely rely on more predictability of genetic modifications on fatty acid and oil metabolism in seeds and other target tissues of crop hosts by use of techniques, such as mass spectrometry-based lipidomics that was essential for optimizing LC-PUFA engineering in camelina seeds, as described by Ruiz *et al.* [19^••^]. Similarly, emerging techniques such as matrix-assisted laser desorption/ionization-mass spectrometry imaging (MALDI-MSI) as applied recently to engineering of oil pathways in Camelina seeds [45^•^] and tobacco leaves [32] are providing insights into spatial heterogeneity of fatty acid compositions in specific lipid classes among cell types in target tissues to enable for more informed metabolic engineering. Ultimately, the task of integrating a small number of transgene-derived activities with a much greater number of endogenous metabolic processes still remains an exciting challenge.

## References and recommended reading

Papers of particular interest, published within the period of review, have been highlighted as:

• of special interest
•• of outstanding interest
